# Relationship of the Temperature-Humidity Index (THI) with Ovarian Responses and Embryo Production in Superovulated Thai-Holstein Crossbreds under Tropical Climate Conditions

**DOI:** 10.3390/vetsci8110270

**Published:** 2021-11-08

**Authors:** Ruthaiporn Ratchamak, Thanaporn Ratsiri, Rujira Chumchai, Wuttigrai Boonkum, Vibuntita Chankitisakul

**Affiliations:** 1 Department of Animal Science, Faculty of Agriculture, Khon Kaen University, Khon Kaen 40002, Thailand; ruthaiporn.kung@gmail.com (R.R.); thanaporn.r@kkumail.com (T.R.); rujira.chumchai@kkumail.com (R.C.); wboonkum@gmail.com (W.B.); 2 Network Center for Animal Breeding and Omics Research, Faculty of Agriculture, Khon Kaen University, Khon Kaen 40002, Thailand

**Keywords:** heat stress, superovulation, threshold point

## Abstract

Heat stress strongly negatively affects reproductive traits in dairy cattle. The purpose of the present study was to investigate the effect of heat stress in superstimulated Thai-Holstein crossbreds under tropical climate conditions. Data included 75 records from 12 superovulated Thai-Holstein crossbreds between 2018 and 2020. Cows were superstimulated with conventional treatment. The mean temperature-humidity index (THI) was evaluated for three data collection periods: during the 9, 21 and 42-day periods before the insemination day to determine the period in which THI mostly affected superstimulation responses. The THI levels/thresholds were determined and interpreted together with the superovulatory response. A significantly negative correlation was obtained for the THI during the period 9 days before insemination. Negative effects on the number of large follicles and corpora lutea began at a THI of 72 and were apparently severe after a THI of 77, similar to the ovulation rate, fertilized ova and transferable embryos (*p* < 0.05). Meanwhile, more degenerated embryos were found with THI values (*p* < 0.05). The superovulatory response in Thai-Holstein crossbreds under tropical climate conditions is highly affected by heat stress starting at a THI of 72 and becomes more severe at a THI higher than 77.

## 1. Introduction

Heat stress is such an important issue for dairy production because of its negative effects not only on depressed milk production [1] but also on reduced reproductive performance [2,3]. Direct effects of thermal discomfort in summer on fertility have been described in several reviews [2,4,5]. Heat stress seems to modify the efficiency of folliculogenesis and to have negative effects on follicle quality [6]. In vitro cultures have been used to study the adverse effects of elevated temperature on incomplete oocyte maturation, fertilization and early embryonic development. The direct exposure of oocytes at the germinal vesicle stage to 41 °C for 6–12 h showed reduced ability to undergo nuclear maturation and embryo development [7]. Pre-incubation of sperm at 40.0–42.0 °C for 4 h decreased sperm motility and integrity and increased sperm damage [8]. Meanwhile, the heat sensitivity of embryos is considered to be stage-dependent, in which early-stage embryos are more susceptible to elevated temperatures than advanced-stage embryos, such as morulae or blastocysts [9,10,11]. These effects result in low fertility during the hot season.

Therefore, all the biological and technical causes for the failure of a female to produce a blastocyst 7 day after natural or artificial insemination (AI) are avoided when a blastocyst is transferred into the female [12]. The embryo transfer has been used instead of AI to improve pregnancy rates during the summer season, as those transferrable blastocysts have a higher heat resistance compared with either ovulated oocytes or earlier-stage embryos [13]. However, it is noted that to have satisfactory pregnancy results, in vivo-derived embryos should be collected from non-heat stressed donors [14].

The temperature-humidity index (THI), representing the combined effects of environment temperature (°C) and relative humidity (%), is widely used to measure the impact of heat stress on dairy cattle. A negative correlation exists in cattle on reproduction traits when the THI crosses a threshold level. The conception rate in dairy cows declines with the threshold points of THI at 72–73 [15,16]. Some studies have inferred that cows exposed to chronic heat stress for at least two estrous cycles showed impaired conception rates [16], as the growth of small antral follicles to preovulatory follicles in the cows requires about two estrous cycles [17]. Meanwhile, some studies referred to the period of one estrous cycle as able to bring about reduced follicle growth and incomplete dominant follicles [6], subsequently resulting in a decreased conception rate [16]. However, there are currently no studies that determine the duration of heat load and THI levels that affect the superovulatory responses in cattle.

The purpose of the present study was therefore to investigate the effects of heat stress for superovulation parameters in crossbred Thai-Holstein dairy cows under tropical climate conditions. Specifically, (1) to identify periods of heat stress which are closely relative to the day of insemination associated with ovarian responses, (2) to estimate heat stress (THI) levels/thresholds based on large follicles responses and (3) to examine the effect of heat stress levels on ovarian responses and embryo production in Thai-Holstein crossbreds.

## 2. Materials and Methods

### 2.1. Animal Care

Data included 75 records from 12 superovulated Thai-Holstein crossbred cows between 2018 and 2020. All of the cows were nonlactating, 3 to 6 years of age and had body condition scores (BCS) between 3 and 3.5 (1–5 scale). They were maintained in straw and fed twice daily with a total mixed ration formulated based on requirements for Holstein cows [18]. All cows had free access to water and mineralized salt. All animal procedures were approved by the Institutional Animal Care and Use Committee of Khon Kaen University, Thailand (Reference No. 660201.2.11/274 (52)).

### 2.2. Superovulation Treatment and Embryo Recovery

**Figure 1:**
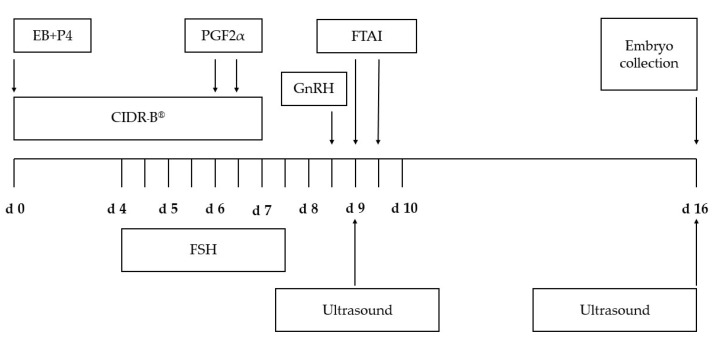
Superovulation treatments in the experiment. CIDR-B^®^, intravaginal device impregnated with 1.56 g progesterone. EB, 5 mg of estradiol-17β. FSH, 400 mg of follicle-stimulating hormone in eight decreasing doses. P4, 50 mg of progesterone. PGF_2α_, 25 mg of *prostaglandin* F_2α_. GnRH, 0.01 mg of Gonadotrophin-releasing hormone. FTAI, fixed time artificial insemination.d, day of superovulation treatment.

The superovulation treatment was induced according to (Figure 1). On a random day of the estrous cycle (Day 0), the estrous cycles were synchronized by an intravaginal device impregnated with 1.56 g progesterone (Eazi-Breed CIDR-B^®^, Zoetis Animal Health, Kalamazoo, MI, USA) and a single dose intramuscular (IM) injection of 5 mg estradiol-17β plus 50 mg progesterone (SRC Animal Health, Pak Chong, Nakhon Ratchasima, Thailand) to induce follicular regression and emergence of a new follicular wave. The superovulatory treatment was initiated on Day 4; cows received 400 mg of FSH (Folltropin^®^-V, Bioniche Animal Health, Belleville, ON, Canada) given IM twice daily, in a decreasing dose (80, 80, 60, 60, 40, 40, 20, 20 mg, respectively) over 4 days. In the morning and evening of Day 6, 25 mg of PGF_2α_ (Lutalyze^®^, Zoetis Animal Health, Kalamazoo, MI, USA) was administrated IM, and CIDR-B^®^ were removed on the morning of day 7. Cows received 0.01 mg of GnRH (Receptal^®^, MSD, Unterschleissheim, Germany) by IM to induce ovulation in the evening of Day 8 and were artificially inseminated 12 and 24 h later. Ova/embryos were collected 7 days after insemination (Day 16) using a nonsurgical technique to flush the uterine horns as described by Ratsiri et al. [19], evaluated and classified for quality according to the criteria of Lindner and Wright [20]. The numbers of ova and embryos were recorded. The percentage of fertilize ova was calculated from the number of fertilized ova of the total number of ova/embryos recovered. Only embryos that graded A and B were considered transferable while those that were degenerated were classified as degenerated embryos. The percentages of transferable embryos and degenerated embryos were calculated based on the total number of fertilized ova/embryos as reported previously [21].

### 2.3. Ultrasound Examination

The ovaries were examined two times by transrectal ultrasonography (HS-2000 ultrasound scanner; Honda Electronics Co., Toyohashi, Japan). Firstly, on Day 9 (before insemination) to count the number of large follicles (≥10 mm), and secondly, immediately before embryo collection on Day 16 of the program to count the number of corpora lutea (CL) and unovulated follicles (≥9 mm). The ovulation rate was calculated by dividing the number of CL by the number of ovulatory follicles [22].

### 2.4. Temperature-Humidity Index (THI)

**Figure 2:**
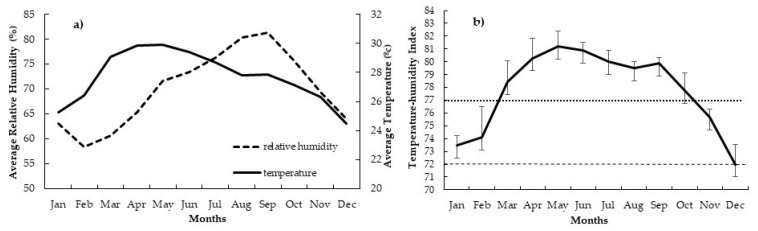
Average of relative humidity and temperature (**a**) and temperature-humidity index (**b**) from 2018 to 2020. (-----; a THI of 72), (▪▪▪▪▪; a THI of 77).

Climate data were obtained from the weather station closest to the dairy farm (3 km distance). The weather information included daily temperature, and relative humidity recorded every 3 h, which were used to calculate the THI between 2018 and 2020, as presented in Figure 2. Meanwhile, those measures of THI in the farm area were recorded with the same frequency using an automatic temperature and humidity meter (data logger; EL-USB-2). The THI was calculated according to the National Oceanic and Atmospheric Administration [23]; THI = (1.8 × temp + 32) − (0.55 − 0.0055 × RH) × (1.8 × temp − 26), where THI was the temperature and humidity index, temp is the temperature (°C), and RH is the relative humidity (%).

To determine which period of heat stress was mainly correlated with the superovulatory responses, the means for THI in the farm area during three different periods relative to the day of insemination were collected and compared with the superovulatory responses. Period 1 was collected during 9-day period (starting at the time of estrus synchronization: Day 0 of superovulatory treatment) to insemination day as a new follicular wave was induced. Periods 2 and 3 were collected during 21 and 42-day periods before the insemination day (one to two estrous cycles, respectively) [15,16]. Then, the THI during the period that heat stress was mostly correlated with the superovulatory responses, was used to determine the threshold of heat stress and to study the relationship between the THI and superovulation responses. The THI levels that affected the superovulatory response were determined based on visual inspection of the graph’s broken arrow point and the coefficient of determination (R^2^) of the graph on the number of large follicles parameter, which is the most critical parameter that indicated the effect of response to the superovulation treatment [24].

### 2.5. Statistical Analysis

The correlation between THI during insemination and the superovulatory responses was performed using the Pearson correlation method by “Proc corr” in the SAS program. The appropriate insemination day was selected for comparative analysis of the THI level on the superovulatory responses. Two methods are used to determine the threshold point of heat stress: (1) The visualisation technique was used to determine the heat stress level by graphing the relationship between THI values and the superovulatory responses and considering the graph’s broken arrow point. (2) The accuracy of the prediction equation by THI level depends on the coefficient of determination (R^2^), which can be calculated from the equation:

(1) $$R^{2}=1-\frac{\mathrm{SSE}}{\mathrm{SST}}$$

where SSE is the sum of squares error and SST is the sum of squares total. At the same time, the influence of the THI level on superovulatory response parameters was also tested with a simple nonlinear regression analysis and correlation analysis (correlation coefficient; r-value). Then, an estimated regression equation was constructed based on the regression equation that best fit the data.

## 3. Results

The mean temperature and relative humidity were recorded from 2018 to 2020; averages were 27.68 °C and 69.94%, respectively. As shown in Figure 2a, the monthly average temperature was lowest in December (24 °C) and was highest in April and May (30 °C). The average relative humidity was lowest in March (60%) and was highest in September (81%). Figure 2b shows the minimum, maximum and average for THI of each month, where the average THI was lowest in December (72) and highest in May (81).

### 3.1. Correlation between THI during Periods of Heat Stress Relative to Insemination Day (9, 21 and 42 Days before Insemination) with the Superovulatory Responses

The correlations between the THI during the 9, 21 and 42-day periods before the insemination day (three periods) with the superovulatory responses in terms of the number of large follicles, number of CL, number of unovulated follicles and ovulation rate in Thai-Holstein crossbred cows are shown in (Table 1). A negative correlation was obtained in all three periods on all parameters of superovulatory responses except ovulation rate. However, those of significantly negative correlation were only for THI during the period 9 days before insemination (number of large follicles: −0.38, *p* = 0.04; number of CL: −0.40, *p* = 0.03). The increase in the THI value led to the decrease in the number of large follicles and the number of CL of 0.38 and 0.40 per THI unit, respectively. Therefore, the THI during the period 9 days before insemination was selected to study the relationship between THI and superovulatory responses.

### 3.2. Determination of Heat-Stress Threshold and Relationship between THI and Superovulatory Responses

**Figure 3:**
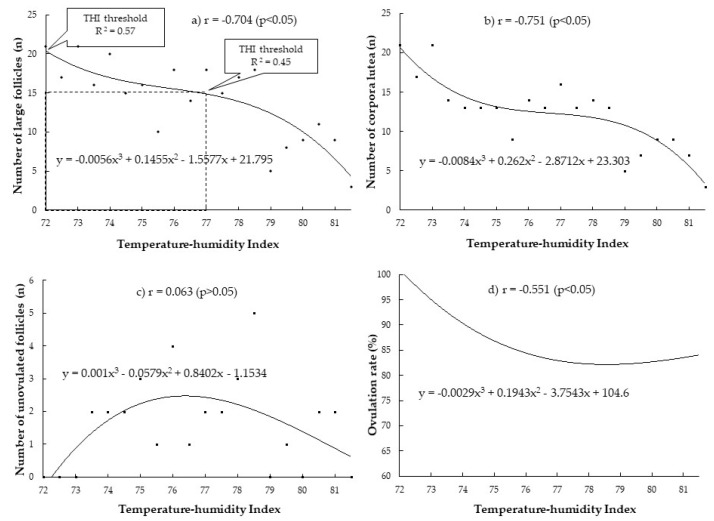
Relationship between THI during the period 9 days before insemination and superovulatory response in terms of number of large follicles (**a**), number of corpora lutea (**b**), number of unovulated follicles (**c**) and ovulation rate (**d**) in Thai-Holstein crossbred cows.

Based on the broken arrow point on the graph and R^2^ regarding the number of large follicles as the main parameters (see Figure 3a), there was a significant decline in the number of large follicles starting at a THI of 72, with the highest R^2^ (0.57) and a more severe decline after a THI of 77 (R^2^ = 0.45). The correlation coefficient (r-value) was negative for the number of large follicles (r = −0.704). In other words, the number of large follicles decreased with the increase of THI.

The numbers of CL and the ovulation rate were significantly affected; the r-values were −0.751 and −0.551, respectively (Figure 3b*,*d). Meanwhile increasing the THI had no significant effect on the number of unovulated follicles; the r-value was 0.063 (Figure 3c).

**Figure 4:**
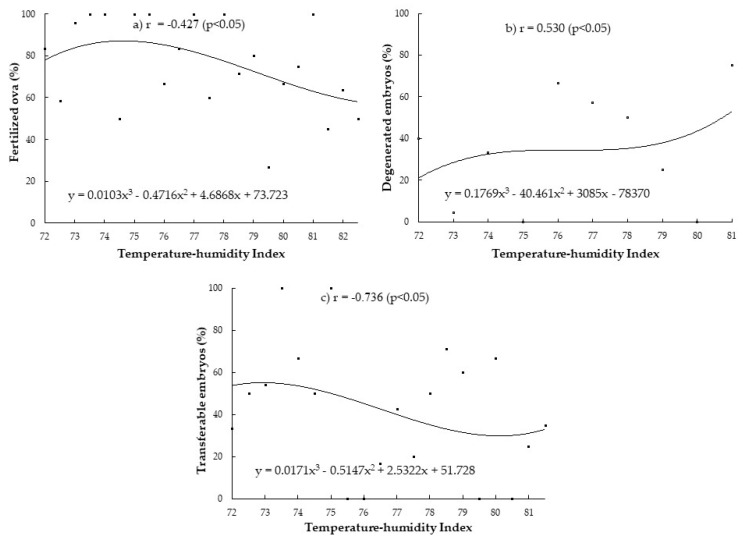
Relationship between THI during the period 9 days before insemination and embryo quality in terms of fertilized ova (%) (**a**), degenerated embryos (%) (**b**), and transferable embryos (%) (**c**) in Thai-Holstein crossbred cows.

The relationship of THI during the period 9 days before insemination with embryo quality is shown in Figure 4. The percentages of fertilized ova and transferable embryos significantly decreased (r-values = −0.427 and −0.736) (Figure 4a*,*c). Meanwhile more degenerated embryos were found while THI increased (r-value = 0.530; *p* < 0.05; Figure 4b).

The regression equation that best fits the present data was y = b_1_X^3^ + b_2_X^2^ + b_3_X + a. This equation form is a third-order degree polynomial nonlinear regression. This equation was used in every parameter. However, the difference regression coefficients (b_1_, b_2_, and b_3_) and y-intercept (*a*) were variable depending on each parameter’s x and y values. The final models utilized to generate the regression lines specific to each parameter are demonstrated in Figure 3 and Figure 4.

## 4. Discussion

In the present study, we evaluated the impact of heat stress as measured by THI on superovulation parameters in Thai-Holstein crossbreds under tropical climate conditions. The mean of the THI during the period 9 days before insemination was used to assess the risk of heat stress. A significant negative correlation between THI and ovarian follicle responses in terms of numbers of large follicles and CL that decreased at a THI of 72 and a more severe decline after a THI of 77 subsequently affected the lower ovulation rate, fertilized ova, and transferable embryos. Meanwhile, significantly more degenerated embryos were found with increasing THI.

The average THI of the three data collection periods negatively correlates with the superovulatory response in terms of the number of large follicles and CL. It has been demonstrated that cows exposed to longer heat stress showed a largely decreased activity of granulosa cells and androstenedione production in the large follicles, which is necessary for follicular growth [25]. However, the THI during the period 9 days before insemination is significantly affected compared with the other periods. Some studies inferred that cows exposed to chronic heat stress for at least two estrous cycles showed impaired conception rates [16], as the growth of small antral follicles to preovulatory follicles in the cows requires about two estrous cycles [17]. Meanwhile, Schuller et al. [15] found that the decrease in conception rate to 16% occurs when cows experiencing heat stress during the three weeks before the day of breeding or one cycle of estrous. The effect of heat stress on the beginning of the estrous cycle reduces follicle growth and incompletely dominant follicles [6]. However, in the present study, exogenous hormones were administered to induce a new follicular wave in our superovulation treatment, suggesting that the period from starting synchronization in the superovulation procedure (9 days in our study) could be a critical period to consider heat load that affects to superovulatory response.

A THI account is a useful and easy way to assess the risk of heat stress on production. Many studies have divided different THI ranges according to either milk production [26,27] or fertility traits [28]. However, the heat stress levels that affected different dairy herds were not similar and depended on breed, heat tolerance, temperature, and humidity [29,30]. In Thailand, which is a tropical country, the effect of THI values was studied only for milk production [31,32]. The THI values that exceeded a threshold of 74 seem to decrease milk production, while the THI threshold of 80 represented severe heat stress in Thai-Holstein crossbreds. In any case, the effect of THI in the reproductive traits of Thai-Holstein crossbreds has been little explored. The average open day of Thai-Holstein crossbreds increased if THI was higher than 77 [33]. The season of calving influenced the open day of Thai-Holstein crossbreds [31,34]. In the present study, the declining of superovulation responses in terms of large follicles and CL started at a THI of 72; however, there was a more severe decline after a THI of 77, which is higher than in previous studies on conception rates (at THI 70–75) [15,16,35,36]. These different THI thresholds might result from Thai-Holstein crossbreds having inherited genes from Bos indicus that help them adapt to heat tolerance; therefore, the threshold point of the THI of heat stress on the reproductive traits in Thai-Holstein crossbreds was higher than other breeds. In addition, it is noted that the THI threshold was lower in our study (reproductive trait) compared with previous studies on milk production traits in Thai-Holstein crossbreds [31,32], suggesting that the reproductive traits had a lower tolerance than production traits [37].

GnRH treatment during estrus has been well documented to either prevent ovulation failure or reduce any variation in the ovulation interval by inducing an LH peak [38,39]. Chankitisakul et al. [21] observed a decline of unovulated follicles and increased ovulation rates if GnRH was administrated before insemination. Indeed, we supposed that ovulation failure should not occur with hormone stimulation. However, the ovulation failure with THI increases was presented even when ovulation was induced with GnRH treatment in the protocols of our study. Similarly to a previous study, more decreasing ovulation rates were found in warm periods than in cool periods [40]. It is therefore inferred that heat stress could increase the proportion of cows that fail to ovulate even when GnRH was administered at estrus to promote ovulation.

The reduction in fertilization rate and increased embryonic mortality were observed in heat-stressed dairy cows [41,42]. Similarly, to our results, the percentages of fertilized ova and transferred embryos significantly decreased at moderate and severe heat stress compared with mild heat stress meanwhile the more significant degenerated embryos were found in severe heat stress. A lower fertilized ova might be explained by heat exposure during oocyte maturation. In vitro studies indicated that exposure of culture oocytes to physiologically relevant heat shock (41 °C) during the first 12 h. of maturation decreased their cleavage rate and blastocyst rate by 30%–60% [7,43,44]. Meanwhile, the heat sensitivity of embryos is considered stage-dependent in which early-stage embryos are more susceptible to elevated temperatures than advanced-stage embryos such as morulae or blastocysts [9,10,11]. Excessive reactive oxygen species could reduce the embryo development rate and increase the number of apoptotic cells in embryos cultured in vitro [45]. A higher incidence of degenerated embryos was demonstrated in superovulated cows exposed to heat stress from the period of onset of estrus until embryo development [46].

Considering the year-round THI values of Thailand (Figure 2b), it was evident that the average THI was above 72 from most of the year. Further, a more severe THI of more than 77 was unsuitable for superovulation treatment for almost 8 months. We suggest that the most suitable period for superovutation procedure to realize more large follicles and transferable embryos in Thailand is during December and January when the average THI is 72 to 73. In the previous study in Thailand, Kaewlamun et al. [34] report that the THI was lowest in December (72) and the highest in April (80). A high ratio of cows calved with successful breeding in December/January due to the THI being low. Accordingly, the average milk production was associated with seasonal variations in Thailand, while the THI was lowest in December and January (73) and highest in April (82) [32]. Moreover, farm management is vital to embryo production on superovulated cows, and the influence of heat stress should be resolved.

## 5. Conclusions

The periods from starting at the time of estrus synchronization, superstimulation to insemination (total = 9 days in the present study) are important for considering the heat load that could affect the superovulatory response. The decline in superovulatory response starts at a THI of 72 and becomes more severe at THI values higher than 77.

## Figures and Tables

**Table 1:** Correlation between THI during periods of heat stress relative to insemination day (9, 21 and 42 days before insemination) with the superovulatory responses in Thai-Holstein crossbred cows.

Periods of Heat Stress before Insemination	Large Follicles (Number)	Corpora Lutea (Number)	Unovulated Follicles (Number)	Ovulation Rate (%)
THI 9-day	−0.38	−0.40	−0.27	0.15
*p*-value	0.04 *	0.03 *	0.21	0.46
THI 21-day	−0.32	−0.35	−0.32	0.14
*p*-value	0.09	0.07	0.14	0.50
THI 42-day	−0.24	−0.27	−0.30	0.16
*p*-value	0.21	0.16	0.16	0.46

* *p* < 0.05.

## Data Availability

The data are available upon request of the corresponding author.
